# Supplementary material for: A symbiotic MLO gene regulates root development via RALF34-triggered Ca2+ signaling in Lotus japonicus
Source: Plant Physiol. 2026 Jul 9;201(3):kiag485. doi: 10.1093/plphys/kiag485 (PMC13418360; doi:10.1093/plphys/kiag485)
Supplement: kiag485_Supplementary_Data [file kiag485_supplementary_data.zip › Binci_et_al_Supplementary_Tables_S2-S3-S4.pdf]

Supplementary Table S2. Sequences of LjRALF mature peptides and *LjMLO4* optimized coding sequence used for synthesis.

LjRALF34	FWRRVKYYISYGALSANRIPCPPRSGRSYYTHDCYKARGPVHPYSRGCSIITR<br>CRR
LjRALF33	ATTKYISYGSLRKNTVPCSRRGASYYNCRTGAQANPYNRGCSAIARCGLYS
LjMLO4 codon optimized coding sequence fused to HIS-tag and STREP-tag at the 3’ end	ATGGCCGGTCCTGCATTAGGCGAGCGTTCATTACAGGAAACCCCTACCTG<br>GGCGGTGGCAGCAGTTTGCGCCGTTTTCATAATTGTGTCAGTACTGATAG<br>AGCACGGCATTGACTCGCTGGGTGAATGGTTCCATAAGCGTCACAAGAA<br>GGCAATGTCAGAGGCACTCGAAAAGATAAAGGCCGAACTGATGTTGCTG<br>GGCTTTATCTCTTTACTCCTGACCTTTGGAACGAAGTACATCGCGAAAAT<br>TTGTATACCGGTCAACGTGGGCGACACAATGCTGCCCTGTAACAAAGCTC<br>TCTTAAAGGTGGACGATAGTAAGGACGACCGCCGGCGACTCTTAAGTTTT<br>GACGAGAACGTCGTTTGGCGAAGAGGATTGGCCGCCGCTAGCGGCGACG<br>ATTACTGCCTGTCTAAGGGTAAGGTACCGCTTATTTCCCAGACGGGCGTA<br>CATCAGCTTCACATCTTCATCTTTGTATTAGCTGTGTTTCATATCTTTTATT<br>CGGTAATGACAATGGTACTTGCTCGCGCGAAGATGCAACGCTGGAAGAA<br>CTGGGAGCAGGAGACGTCGAGCCTCGAATACCAGTTCACGAACGACCCG<br>GCGCGATTTCGGTTCGCACATCAGACCACTTTTGTGCGACGCCATAGCGG<br>TTGGACCCGTAAGCCGGGGATCCGCTGGATCGTCGCTTTCTTTCGTCAGT<br>TCTTCGCATCAGTATCAAAAGTTGACTATATGACGATGCGTCACGGTTTC<br>ATCAATGCGCACTTCAACCCGGACTCCAAGTTCGATTTCCACAAGTATAT<br>TAAGCGCAGCATGGAAGACGACTTCAAGGTAGTTGTGGGCATTTCATTAC<br>CCCTCTGGGCATTCGCTATTATATTTATGCTCTTAAATATCCAAAAGTGGT<br>ATACCCTTTCATGGTTGAGTCTTGCTCCGCTGGTTATTCTGCTGTTGGTTG<br>GCACAAAATTGGAGCTGATTATAATGGAGATGGCGCAGGAGATTCAGGA<br>CCGCACCACCATAGTCCGTGGTGTGCCTGTTGTGGAACCTAATAATAAAT<br>ACTTTTGGTTCAACAAACCCGAGTGGATACTGTTTCTGATTCACTTCACTC<br>TCTTCGAAAACGCTTTTCAGATCGCCTACTTTCTCTGGTCTTGGTACGAAT<br>TTAAAATTACCAGTTGTTTTCACGCGGATTTAGCCTTGACTATCACCCGCG<br>TGGTTCTGGGAGTTGCGCTGCAGGTGCTGTGTAGCTATATAACCTTCCCA<br>CTGTACAGTCTGGTTACCCAAATGGGTAGCCATATGAAGAAGGCGATTTT<br>CGAAGAGCAGACTACTAAAGCCCTGAAGAAATGGCAGAAAGTAGCTAAA<br>GAGAAACGAAAACTGCGAAAGGCGGGCATCGACATACCGAGTCGTTCTT<br>CGGTATCGGTATCTATGTCTGGTGAAACTACGCCGTCTCAGGGTTCTTCTC<br>CCCTGCACCTGTTGCATAAATATAATAAACCGTCCCATATCGATAGCGCG<br>GACCTGTACTCCCCTCGTTCTTACCAGTCAGATACTGAATTCTCTGAGAC<br>GGAAGGTAGTACCCATGAGTTGAACGAGATTAAGCCGACTCACCAGCCG<br>CCGAAGAAAGAGGAGACGCATAACATTGACTTCTCCTTCGATAAACCAG<br>GCTCCCACCACCATCACCATCACTGGAGCCACCCGCAGTTCGAAAAGTAG<br>TAG

Supplementary Table S3. List of primers used in this study.

Name	5’ → 3’ sequence	Description
q81	CAATGTCGCCAAGGCCCATGGTG	qPCR FOR primer for *LjATPase* (Binci et al. 2024)
q82	AACACCACTCTCGATCATTTCTCTG	qPCR REV primer for *LjATPase* (Binci et al. 2024)
q11	GATTCATATCCCTGTTACTT	qPCR FOR primer for *LjMLO4*
q12	TAACCTCCTCCTATCATCT	qPCR FOR primer for *LjMLO4*
PT4F	GTACAATGACCTCATGGTTCT	qPCR FOR primer for *LjPT4* (Volpe et al. 2016)
PT4R	CGTTCATCTCGAAATCCTTATC	qPCR REV primer for *LjPT4* (Volpe et al. 2016)
q41	CACGTTGTTAGGACCCCAAT	qPCR FOR primer for *LjSbtM1* (Pimprikar et al. 2015).
q42	TTGAGCAGCACCCTCTCTATC	qPCR REV primer for *LjSbtM1* (Pimprikar et al. 2015)
P2	CCATGGCGGTTCCGTGAATCTTAGG	LORE1 insertion REV
G28	TTCGCTCATGGCCTTCTTATGGCG	*mlo4-1* FOR
G29	AAAGAGGCTTGTTACGGTATCGGCAG	*mlo4-1* REV
G67	TCGTTAGGCGTCACTCAGGCTGGA	*mlo4-3* FOR
G81	TGCTATCAGGATTAAAATGTGCCTGCAT	*mlo4-3* REV
Ubi_F	ATGCAGATCTTCGTCAAGACCTT	LjUbiquitin10 FOR
Ubi_R	ACCTCCCCTCAGACGAAG	LjUbiquitin10 REV
C16	AACAGGTCTCAACCTGGGTAGAGTTATGTCTA ACTCACTG	modA_MLO4_promoter for
C17	AACAGGTCTCATGTTGTTAATTTTGCTCTCAGC TTCTCC	modA_MLO4_promoter rev
C155	AACAGGTCTCAACCTGTCAAACCGCGCCTAAC ATTT	pMLO4(-2000-208)_modA_for
C156	AACAGGTCTCATGTTTTTACAAGTTAATTTATT GTTTTAGAATTTTTCTTAAACA	pMLO4(-2000-208)_modA_rev
C157	AACAGGTCTCAAACATTATATTAATTAATCAAT TATGTCTTGT	pMLO4(-186-1)_ modB_for

C158	AACAGGTCTCAAGCCGTTAATTTTGCTCTCAGCTT	pMLO4(-186-1)_ modB_rev
C159	AACAGGTCTCAAACAGCATATGCTTATATTAATTAATCAATTATG	pMLO4(-AW-Box)_ modB_for
C160	AACAGGTCTCATGTTCGGCCTATGCAAGGTTTACAAG	pMLO4(-2000-208+AW-box)_ modB_rev
C239	ACCGCTGAGCAATAAC	pRham linearization FOR
C240	ATGTATATCTCCTTCTTATAGTTAAAC	pRham linearization REV
C243	TATAAGAAGGAGATATACATATGGCCGGTCCTGCATTAGG	LjMLO4_optimised linearization FOR
C244	GCTAGTTATTGCTCAGCGGTCTACTACTTTTCGAACTGCGGGTGGCTC	LjMLO4_optimised linearization REV
C285	TGGTGATGGTGGTGGGAGCCACCGTGACGCATCGTCATATAG	LjMLO4Δ_optimised linearization FOR

Supplementary Table S4. List of plasmids used in this study

Name	Type	Resistance	SOURCE	Description
pGGA000	entry	ampicillin and chloramphenicol	Addgene - GreenGate cloning system	Empty entry vectors (ccdB+) - ModA
pGGA006	entry	ampicillin	Addgene - GreenGate cloning	Arabidopsis UBQ10 promoter - Module A
GG59	entry	ampicillin	Binci *et al.* 2024	*L. japonicus* 2200bp UBQ10 promoter - Module A
GG83	entry	ampicillin	This study	2000bp promoter of LjMLO4 - ModA
GG148	entry	ampicillin	This study	pMLO4_modA_-2000_-208 - modA
pGGB000	entry	ampicillin and chloramphenicol	Addgene - GreenGate cloning system	Empty entry vectors (ccdB+) - Module B
pGGB003	entry	ampicillin	Addgene - GreenGate cloning	Dummy – module B
GG145	entry	ampicillin	This study	pMLO4_minusAW-box_modB_-1_-224 - ModB
GG146	entry	ampicillin	This study	pMLO4_minusAW-box_minusP1BS_modB_-1_-208 - ModB
GG147	entry	ampicillin	This study	pMLO4_plusAW-box_modA_-2000_-208 - modB

pGGC051	entry	ampicillin	Addgene - GreenGate cloning system	*UidA* gene for GUS staining - modC
pGGD002	entry	ampicillin	Addgene - GreenGate cloning system	Dummy – module D
pGGE009	entry	ampicillin	Addgene - GreenGate cloning system	Arabidopsis UBQ10 terminator - Module E
GG69	entry	ampicillin	This study	Transformation marker cassette: pAtUBQ10::GFP - modF
GG108	destination	kanamycin	Binci et al. 2024	Empty destination vector (ccdB+)
GG62	expression	kanamycin	Binci et al. 2024	cytosolic YFP-linker-aequorin under the control of the LjUBQ10 promoter. Transformation marker: cytosolic mCherry. Destination vector: GG108.
GG88	expression	kanamycin	This study	GUS under p*MLO4.* Transformation marker: cytosolic GFP. Destination vector: GG108.
GG142	expression	kanamycin	This study	GUS under p*MLO4*ΔP1BSΔAW-box. Transformation marker: cytosolic GFP. Destination vector: GG108.
GG143	expression	kanamycin	This study	GUS under p*MLO4*ΔP1BS. Transformation marker:

				cytosolic GFP. Destination vector: GG108.
GG144	expression	kanamycin	This study	GUS under p*MLO4*ΔAW-box. Transformation marker: cytosolic GFP. Destination vector: GG108.
pRham	destination	kanamycin	Lucigen (Middleton, WI, USA)	Empty destination vector - pRham
pRham-LjMLO4	expression	kanamycin	This study	LjMLO4-HIS optimized for *E. coli* expression. Rhamnose-inducible expression
pRham-LjMLO4Δ	expression	kanamycin	This study	LjMLO4Δ-HIS optimized for *E. coli* expression. Rhamnose-inducible expression
pACYC Duet-1(HA1-Aeq)	expression	chloramphenicol	Teardo et al. 2019	Aequorin with IPTG-inducible expression
